# Activity-Based Proteomics Reveals Heterogeneous Kinome and ATP-Binding Proteome Responses to MEK Inhibition in KRAS Mutant Lung Cancer

**DOI:** 10.3390/proteomes4020016

**Published:** 2016-04-27

**Authors:** Jae-Young Kim, Paul A. Stewart, Adam L. Borne, Bin Fang, Eric A. Welsh, Yian Ann Chen, Steven A. Eschrich, John M. Koomen, Eric B. Haura

**Affiliations:** 1 Department of Thoracic Oncology, H. Lee Moffitt Cancer Center and Research Institute, Tampa, FL 33612, USA; jae-young.kim@moffitt.org (J.-Y.K.); paul.stewart@moffitt.org (P.A.S.); adam.borne@moffitt.org (A.L.B.); 2 Proteomics Core, H. Lee Moffitt Cancer Center and Research Institute, Tampa, FL 33612, USA; bin.fang@moffitt.org; 3 Cancer Informatics Core, H. Lee Moffitt Cancer Center and Research Institute, Tampa, FL 33612, USA; eric.welsh@moffitt.org; 4 Department of Biostatistics & Bioinformatics, H. Lee Moffitt Cancer Center and Research Institute, Tampa, FL 33612, USA; ann.chen@moffitt.org (Y.A.C.); steven.eschrich@moffitt.org (S.A.E.); 5 Department of Molecular Oncology, H. Lee Moffitt Cancer Center and Research Institute, Tampa, FL 33612, USA; john.koomen@moffitt.org

**Keywords:** adaptive resistance, desthiobiotin-ATP probe, KRAS mutant lung cancer, LC-MS/MS, MEK inhibitor

## Abstract

One way cancer cells can escape from targeted agents is through their ability to evade drug effects by rapidly rewiring signaling networks. Many protein classes, such as kinases and metabolic enzymes, are regulated by ATP binding and hydrolysis. We hypothesized that a system-level profiling of drug-induced alterations in ATP-binding proteomes could offer novel insights into adaptive responses. Here, we mapped global ATP-binding proteomes perturbed by two clinical MEK inhibitors, AZD6244 and MEK162, in KRAS mutant lung cancer cells as a model system harnessing a desthiobiotin-ATP probe coupled with LC-MS/MS. We observed strikingly unique ATP-binding proteome responses to MEK inhibition, which revealed heterogeneous drug-induced pathway signatures in each cell line. We also identified diverse kinome responses, indicating each cell adapts to MEK inhibition in unique ways. Despite the heterogeneity of kinome responses, decreased probe labeling of mitotic kinases and an increase of kinases linked to autophagy were identified to be common responses. Taken together, our study revealed a diversity of adaptive ATP-binding proteome and kinome responses to MEK inhibition in KRAS mutant lung cancer cells, and our study further demonstrated the utility of our approach to identify potential candidates of targetable ATP-binding enzymes involved in adaptive resistance and to develop rational drug combinations.

## 1. Introduction

A cancer cell’s ability to rapidly adapt to targeted reagents is a major hurdle to the success of targeted therapy for cancer treatment. Examples include the compensatory activation of PI3K/Akt following mTOR inhibition driven by upstream receptor tyrosine kinase activation [1,2,3,4] and feedback activation of B- and C-RAF upon MEK inhibition, especially in the context of RAS-driven cancers [5,6]. This adaptive resistance is complicated by the diversity of kinases and enzymes expressed in cancer cells functioning in interconnected networks; thus, systematically examining how cancer cells respond to drugs could offer novel insights into adaptive resistance mechanisms and point toward effective drug combinations [7,8].

Mass spectrometry-based quantitative proteomics has been employed to assess altered signaling networks after drug treatment at a system-wide level. Gary Johnson and his colleagues developed a chemical proteomics method employing multiplexed kinase inhibitor beads followed by mass spectrometry analysis, allowing a system-wide measurement of drug-induced kinase activity/expression. This platform was employed to assess kinome adaptations to kinase inhibitors in multiple cancer models, including triple-negative breast cancer treated with MEK inhibitor selumetinib [9] and HER2-positive breast cancer treated with HER2 inhibitor lapatinib [10]. These studies highlighted drug-induced dynamic kinome reprogramming involving reactivation of co-expressed receptor tyrosine kinases in response to kinase inhibitors. The multiplexed kinase inhibitor bead approach also revealed differential kinome expression/activity between parental and leukemia cells with acquired drug resistance to BCR-Abl inhibitor imatinib [11].

As an alternative approach that has shown utility in charting rapid dynamic responses, profiling drug-induced global phosphoproteome changes has identified key adaptive changes linked to drug resistance. A tyrosine phosphorylation profiling in *DDR2* mutant squamous lung cancer cells treated with its tyrosine kinase inhibitor dasatinib revealed key compensatory receptor tyrosine kinase activations linked to intrinsic drug resistance [12]. This approach also revealed differential drug-induced tyrosine phosphoproteome responses between naïve and drug-resistant EGFR mutant lung cancer cells to EGFR tyrosine kinase inhibitor [13]. Global phosphoproteome (phospho-Ser/Thr/Tyr) profiling showed that ablation of TBK1 expression in KRAS mutant lung cancers leads to compensatory activation of a panel of receptor tyrosine kinases including EGFR and MET [14]. One limitation of phosphoproteomic approaches is the requirement of large amounts of protein lysates (usually ~30–50 mg) and/or peptide fractionation (usually 12 fractions per sample), which restricts the number of samples or conditions to be analyzed in a practical and reasonably economical mass spectrometry experiments. Finally, important information on adaptive responses driven by other ATP-binding enzymes could be missed by focusing solely on phosphoproteomics.

We hypothesized that we could employ another approach to study adaptive resistance and kinase rewiring using a commercially available desthiobiotin-ATP probe (ActivX, Thermo Scientific), which covalently labels conserved lysine residues in or near the ATP-binding pocket of enzymes, including kinases [15]. Peptides containing the labeled lysine residues are then enriched by streptavidin beads, identified and quantitated by liquid chromatography-tandem mass spectrometry (LC-MS/MS). This approach is an alternative way to overcome the aforementioned disadvantages since it requires relatively small amount of samples (1 mg) and requires no fractionation for LC-MS/MS analysis. It allowed us to test how multiple KRAS mutant lung cancer cell lines differentially respond to MEK inhibition and reveal heterogeneous ATP-binding proteome responses from each individual cell line.

Here, we profiled ATP-binding proteome responses to two clinical MEK inhibitors, AZD6244 and MEK162, in the context of KRAS mutant lung cancer. KRAS mutations occur in nearly 30% of non-small cell lung cancers (NSCLC), yet therapeutic targets for these cancers have not been realized. MAPK signaling has been known to be essential for KRAS-induced lung tumorigenesis [16], and pharmacological inhibition of this pathway (e.g., MEK inhibitor) has been attempted to treat KRAS-driven lung cancers. However, significant clinical responses are still lacking, in part due to the cancer cells’ ability to re-activate ERK via feedback activation of RAF [5,6,17,18]. Recent studies indicated that mutational status of tumor suppressors, p53 or LKB1, in KRAS mutant lung cancer could modulate drug responses to MEK inhibitor AZD6244 [19] and immune checkpoint inhibitors [20]. This raises the possibility that heterogeneous adaptive responses could exist in KRAS mutant lung cancer depending on the status of co-mutated tumor suppressors, further complicating the development of a rational co-targeting strategy. For this study, we employed multiple KRAS mutant lung cancer cell lines harboring diverse p53 and LKB1 co-mutations and differential histology (adenocarcinoma and squamous cell carcinoma) to address heterogeneous adaptive responses. Using these two MEK inhibitors allows for filtering and focus on “on target” effects and not just idiosyncratic drug targets.

## 2. Results and Discussion

**Figure 1:**
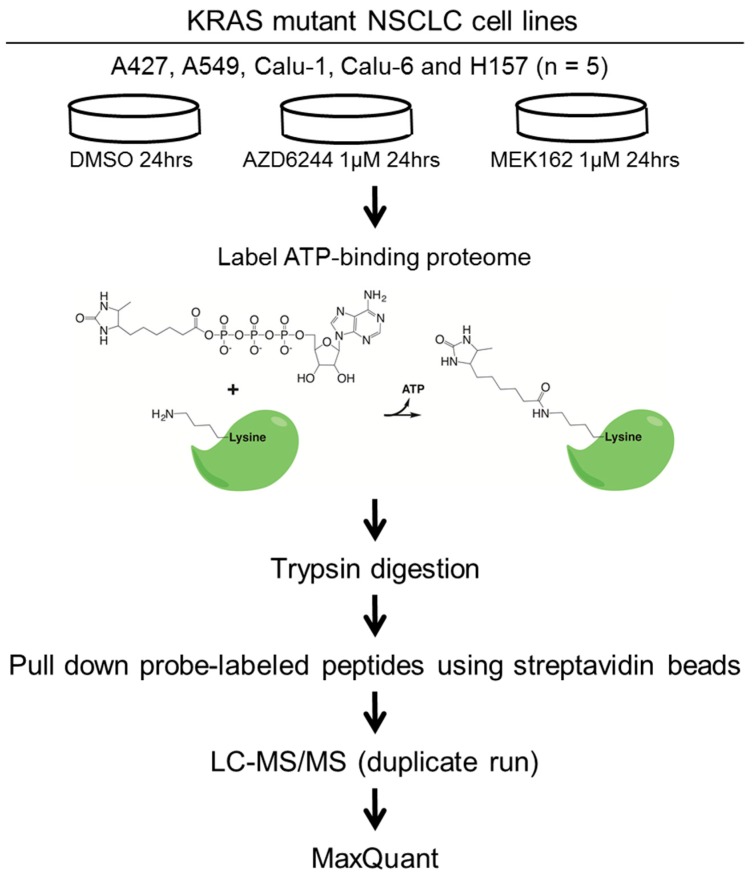
The experimental workflow of this study. Five KRAS mutant NSCLC cell lines harboring differential p53 and LKB1 mutation status are treated with DMSO (vehicle control) or MEK inhibitors (AZD6244 or MEK162). ATP binding proteomes are enriched by the desthiobiotin-ATP probe, followed by identification and quantitation using LC-MS/MS. The raw MS data are processed by MaxQuant software (Version 1.2.2.5) [21].

To address diverse adaptive responses to MEK inhibition in the context of KRAS mutant lung cancer, we employed five KRAS mutant lung cancer cell lines with differential LKB1/p53 mutation status and histology; four lung adenocarcinoma cell lines including A427, A549 (p53 wild type/LKB1 mutant), Calu-1, and Calu-6 (p53 mutant/LKB1 wild type); and a lung squamous cell carcinoma cell line, H157 (p53 mutant/LKB1 mutant). We then assessed how MEK inhibitors remodel their ATP-binding proteomes. Cells were treated with 1 µM of MEK inhibitors (AZD6244 or MEK162) or vehicle control (DMSO), and then ATP-binding proteins were labeled with the desthiobiotin-ATP probe and trypsin digested. We found 1 µM to be a clinically relevant [17] and a 24-h time point was chosen based on a previous study on kinome-level response to a MEK inhibitor [9]. The probe-labeled peptides were enriched by streptavidin beads, followed by LC-MS/MS analysis. The workflow of our study is illustrated in Figure 1.

**Figure 2:**
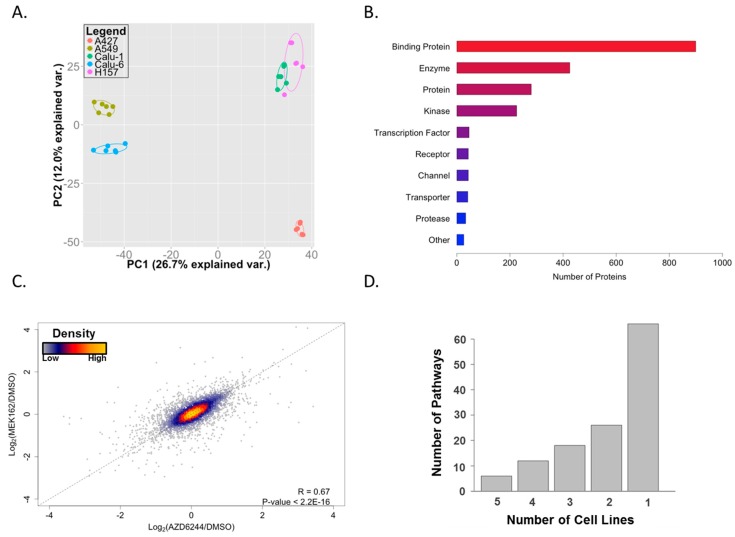
ATP-binding proteomes in KRAS mutant lung cancer cells. (**A**) Principal component analysis (PCA) showing the distinct signature of ATP-binding proteomes on individual cell lines; (**B**) functional annotation of identified proteins using GeneGO. The “Binding Protein” category includes proteins such as ATP-binding proteins, protein complex subunits, and proteins involved in protein-protein interactions; (**C**) scatterplot showing a high correlation of the effect of two MEK inhibitors; and (**D**) unique GeneGO pathway enrichment from altered proteins on individual cell lines. A majority of enriched pathways are observed only in one cell line.

After filtering out low-confidence peptides, we identified 5800 peptides that were associated with 1925 protein groups (Table S1). Principal component analysis (PCA) shows clustering based on cell lines and not for treatment type, suggesting that, despite shared KRAS mutant status, the ATP-binding proteomes and responses to MEKis are cell type specific with the exception of similarity between Calu-1 and H157 in the first two principal components (Figure 2A). Interestingly, the X and Y axes of the PCA plot, corresponding to the first two principal components of the data, are not associated with drug effect on cell viability, which showed a similar reduction across the cell lines (50%–60% reduction at 1 µM; data not shown) nor with co-mutating tumor suppressor (p53 and LKB1) status. This suggests that these are not major factors dictating the behavior of the ATP-binding proteome. However, given the small number of cell lines employed in this study, larger studies with more power are necessary to investigate this further. From these 1925 protein groups, we were able to identify 174 protein kinases or 225 kinases in total (including nine lipid kinases and 42 other generic/small molecule kinases). These results are comparable to previously-reported identifications using this technology (188 protein kinases [22], 136 protein kinases [23], and 41 protein kinases [24]). Although kinases were a primary focus, we were able to identify hundreds of other proteins in a variety of different classes (Figure 2B). We were also able to detect some proteins that have not been reported to bind ATP. These proteins likely received an ATP-probe by being adjacent to a substrate of, or in a complex with, an ATP-binding protein or by promiscuous binding of the ATP-probe during the labeling step of the experimental workflow.

Next, we set out to examine how MEK inhibitors remodel ATP-binding proteomes in each individual cell line. We first averaged the log_2_ fold-changes of drug/control across all cell lines. This allowed us to observe if there were any differences between the effects of the two drugs. Figure 2C shows a strong, positive correlation between the two inhibitors, suggesting that these drugs are behaving in a similar manner. Next, we defined altered peptides as those whose labeling levels (calculated as log_2_(drug/control)) were changed by at least one standard deviation away from the average by both MEK inhibitors (Table S2). In order to gain a comprehensive view of the altered ATP-binding proteome, individual proteins corresponding to these altered peptides were subjected to GeneGO MetaCore pathway enrichment analysis. We observed strikingly diverse enriched pathways; most of the enriched pathways were observed in only one cell line, which highlights a heterogeneous response to MEK inhibition (Figure 2D and Table S3). Representative pathways enriched from each cell line are shown in Table 1. Despite the heterogeneity, cytoskeleton remodeling pathways were highly enriched across all cell lines. We observed enrichment of glycolysis/gluconeogenesis pathways in A427, A549, and Calu-1 cells, suggesting MEK inhibition leads to altered glucose metabolism in these cells. Previous studies indicated that pharmacological inhibition of BRAF or MEK suppresses glycolysis in the context of melanoma cells harboring activating mutation of BRAF [25,26], warranting future studies to examine whether MEK inhibition leads to metabolic perturbation and, if so, to determine its clinical implication on lung cancer.

**Figure 3:**
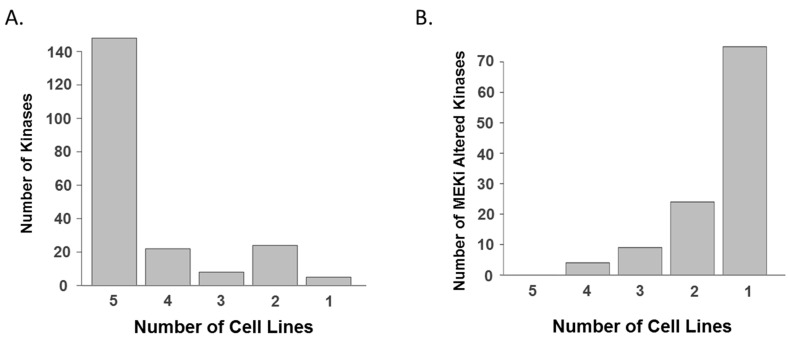
Kinomes in KRAS mutant lung cancer cells. (**A**) Common kinome signature in KRAS mutant lung cancer cell lines based on protein groups. A majority of kinases are identified and quantitated in all five cell lines; and (**B)** unique kinome responses to MEK inhibitors in individual cell lines based on protein groups. A majority of altered kinases, whose drug-induced log_2_-transformed fold changes are greater than one standard deviation, are observed only in one cell line.

**Figure 4:**
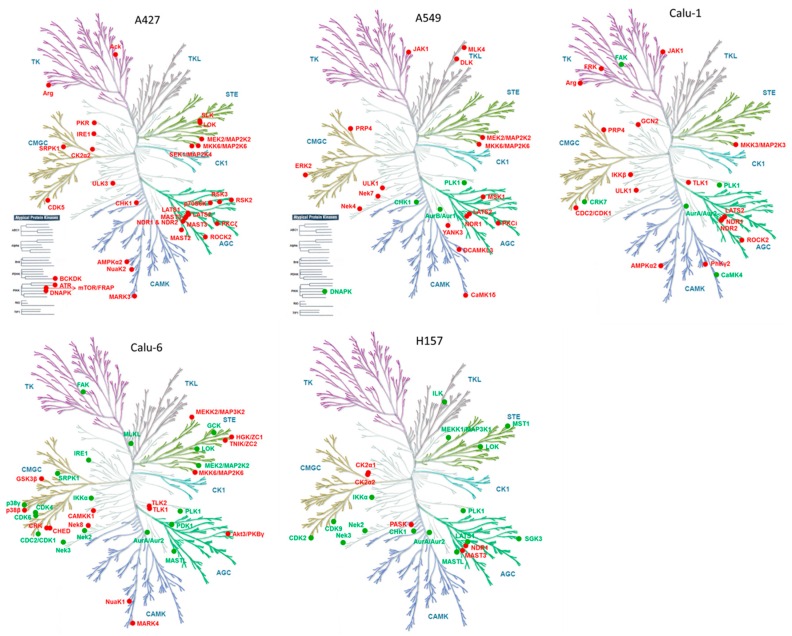
Kinome response to MEK inhibition. Altered kinases from each cell line are mapped on kinome trees using a Web-based kinome render tool [44]. Red and green nodes signify increased and decreased kinases, respectively. Illustrations reproduced courtesy of Cell Signaling Technology.

Next, we focused on specific kinome responses to MEK inhibitors in each cell line given the importance of kinase signaling in regulating cell growth and survival [27]. In total, 225 kinases, which were quantified from the five cell lines, showed quite similar overall expression patterns between cell lines (Figure 3a and Table S4); however, each cell line showed unique drug-induced altered kinase list (Figure 3b and Table S5). This observation prompted us to hypothesize that each cell line could show a distinct kinome response to MEK inhibition, and the trend of changes in kinases is illustrated in kinome trees (Figure 4). Despite this heterogeneity, we observed a number of peptides that were consistently altered by both MEK inhibitors in more than two cell lines, which suggests common adaptive responses could exist (Table 2).

Given the importance of tyrosine kinases in cell growth and survival, we first focused on altered tyrosine kinases (TK group) in each cell line. Despite the well-known role of drug-induced activation of receptor tyrosine kinases in drug resistance, we could not observe significantly-altered receptor tyrosine kinases from our results. However, we found that the non-receptor tyrosine kinase JAK1 is upregulated in A549 and Calu-1. It has been reported that MEK inhibitors induce the JAK-STAT pathway promoting cancer cell invasiveness in melanoma [28], suggesting that MEK inhibitors could induce a similar phenotype in KRAS mutant lung cancer. We also observed downregulation of focal adhesion kinase FAK1 in Calu-1 and Calu-6, suggesting that MEK inhibition could lead to altered integrin signaling and cell motility. Second, MEK inhibitors upregulated stress-activated protein kinase (SAPK) signaling, including MKK6 (A427, A549, Calu-6) and MKK3 (Calu-1), both of which are upstream of p38 MAPK. Third, MEK inhibitors downregulated mitotic kinases PLK1 and its upstream Aurora A kinase (AURKA). Notably, this effect was observed in most of the cell lines, suggesting potential crosstalk between MAPK signaling and cell cycle kinases in the context of KRAS mutant lung cancer. Several preclinical studies have already indicated KRAS mutant cancers are specifically vulnerable to inhibition of mitotic kinases [14,29,30]. However, significant clinical responses of mitotic kinase inhibitors are still lacking in lung cancer [31,32]. It is, thus, possible that combined inhibition of MAPK and mitotic kinases could be synergistic in the context of KRAS mutant lung cancer. Finally, MEK inhibitors upregulated autophagy kinases ULK1 (A549 and Calu-1), ULK3 (A427), and AMPK (A427 and Calu-1). Autophagy is a self-digestion process that is generally activated by nutrient deprivation, but it is also known to be induced by therapeutic stresses in cancer cells, contributing to drug resistance [33,34]. These observations are consistent with previous studies indicating RAF and MEK inhibitors could induce cytoprotective autophagy, leading to drug resistance in the context of BRAF mutant melanoma [35,36] and KRAS mutant lung cancer [37]. Our results reveal kinases that are potentially responsible for autophagy induction in KRAS mutant lung cancer cells treated with MEK inhibitors, further offering potential rational drug combinations.

The desthiobiotin-ATP probe employed in this study was originally developed for drug target profiling to assess the specificity of kinase inhibitors [15,22], but we employed it here to assess global ATP-binding proteome/kinome response to clinical MEK inhibitors in the biological context of various KRAS mutant lung cancer cell lines. Gygi and his colleagues reported that desthiobiotin-ATP probe did not specifically enrich active forms of kinases [38], thus, the changes are likely to be associated with total protein level, rather than activity, at least in the context of kinases. The importance of drug-induced transcriptome and kinome reprograming and its implication in drug resistance is increasingly being recognized [9,10]. In light of these studies, the value added from our study is heterogeneity of drug-induced kinome and ATP binding protein expressions in KRAS mutant lung cancer. Our study also uncovered novel adaptive responses, suggesting that MEK inhibition could lead to metabolic alteration, abnormal mitosis, and induction of cytoprotective autophagy. Our results could be integrated with phosphoproteomics datasets to address how the drug-induced kinome changes lead to modulation of the phosphoproteome. Further, synthetic lethal kinome RNAi screening [39,40,41,42] or pharmacologic vulnerability screens [43] could be combined to assess translational potential of our results.

## 3. Materials and Methods

### 3.1. Cell Lines and Drugs

Cells were maintained in RPMI 1640 medium supplemented with 10% FBS. Cells were confirmed to be free of mycoplasma using PlasmoTest (Invivogen, San Diego, CA, USA). AZD6244 and MEK162 were purchased from ChemiTek (Indianapolis, IN, USA). Drugs were reconstituted with DMSO at 50 mM concentration, and aliquots were stored at −80 °C. 

### 3.2. Drug Treatment and ATP Probe Labeling

Cells at 80% confluence were treated with DMSO (vehicle control) or 1 µM MEK inhibitors (AZD6244 or MEK162) for 24 h; cell pellets were then harvested in ice-cold PBS and stored at −80 °C until probe labeling. Cell lysates were prepared and labeled according to the manufacturer’s recommendations for the Pierce Kinase Enrichment Kits and ActivX Probes (Thermo Scientific, Rockford, IL, USA)). Briefly, cell pellets were re-suspended in 600 µL of Pierce IP lysis buffer and sonicated. The lysates were cleared by centrifugation at 16,000 g for 10 min at 4 °C and desalted by Zeba Spin Desalting Columns (Thermo Scientific, Rockford, IL, USA). The concentration of protein was measured using a Bradford assay, and a total of 1 mg was used for ATP probe labeling. MnCl_2_ was added to the lysate to a final concentration of 20 mM for 10 min, and then the desthiobiotin-ATP probe was added at a final concentration of 5 mM for 15 min. All reactions were performed at room temperature.

### 3.3. MS Sample Preparation and LC-MS/MS Analysis

The probe-labeled lysates were denatured in 5 M urea and 5 mM DDT at 65 °C for 30 min and then reduced in 40 mM iodoacetamide at room temperature for 30 min. The lysates were desalted by Zeba Spin Desalting Columns and then digested with 20 μg/mL of trypsin for 2 h at 37 °C. Desthiobiotinylated peptides were captured by 50 μL slurry of high-capacity streptavidin beads for 1 h. The beads were washed with lysis buffer, PBS buffer, and LC-MS grade water in sequence with four washes for each buffer, and then the peptides were eluted by 50% acetonitrile in 0.1% formic acid (TFA) water. The peptides were lyophilized in a vacuum concentrator and re-suspended in 20 μL of injection buffer containing 2% acetonitrile and 0.1% TFA. The LC-MS/MS analysis was performed as previously described [45].

### 3.4. Data Analyses

The peptides identified in the mass spectrometry data were analyzed and quantified using MaxQuant Version 1.2.2.5 (http://www.maxquant.org) [21]. For the search parameters, desthiobiotin-ATP labeling was selected, enzyme specificity was set to fully tryptic cleavage, allowing a 10 ppm mass error in the main search, a maximum number of three modifications per peptide, and four maximum missed cleavages. Fragment ion tolerance was set to 0.6 Da. Protein and peptide false discovery rates were set to 0.1 and 0.05, respectively, and the minimum peptide length was set to six amino acids. MS/MS data were searched against the UniProt human database combined with common contaminants and concatenated with the reversed versions of all sequences using the Andromeda search engine integrated into MaxQuant. The protein kinases were annotated using GeneGO (Boston, MA, USA) and intensity of each peptide corresponding to protein kinases were used to calculate the inhibition of each drug on the relevant peptide compared with DMSO control group.

Peptide site intensities were extracted from MaxQuant output and input into the Libaffy software package [46]. Libaffy features access to iterative rank-order normalization (IRON), which we have previously used in proteomics applications [47,48]. Peptide site intensities were normalized using IRON before being input into R/RStudio with corresponding metadata [49,50]. Peptides were filtered to remove posterior error probability >0.1, reverse sequences, non-human peptides, and for peptides that were identified but had no corresponding intensities. Intensities were then log_2_-transformed. Concordance of log_2_ ratios between control and treatment pairs was measured using Pearson’s correlation (herein referred to as *R*). GeneGO MetaCore was used to annotate individual proteins from the identified protein groups [51] and used for pathway searches on all proteins in identified protein groups. A protein group was included in the pathway search if there was at least one standard deviation change in the log_2_ ratios of both treatments. Inconsistent log_2_ ratios (*i.e.*, the log_2_ ratio indicates an increase from AZD6244 but a decrease from MEK162) were not included in the pathway analysis.

## 4. Conclusions

Our results demonstrate the utility of the desthiobiotin-ATP probe coupled with LC-MS/MS in assessing adaptive kinome/ATP-binding proteome responses to cancer drugs. This approach could allow mass spectrometry analysis of multiple samples or conditions (e.g., time points) since it does not require a large amount of total protein from each sample and peptide fractionation is unnecessary prior to LC-MS/MS analysis; this approach could be an alternative to kinase inhibitor bead-based kinome profiling and phosphoproteomics. These approaches are complementary and could be combined to identify novel adaptive resistance mechanisms.

## Figures and Tables

**Table 1:** Top five pathways enriched by GeneGO pathway analysis of altered ATP binding proteins on individual cell lines.

**A427**	***p* Value**
LRRK2 in neurons in Parkinson‘s disease	4.62 × 10^−11^
Development_Slit-Robo signaling	3.63 × 10^−10^
Development_Regulation of cytoskeleton proteins	2.76 × 10^−9^
Cytoskeleton remodeling_Regulation of actin cytoskeleton by Rho GTPases	1.53 × 10^−8^
Regulation of CFTR activity (normal and CF)	7.02 × 10^−8^
**A549**	***p* Value**
LRRK2 in neurons in Parkinson‘s disease	1.54 × 10^−13^
Cytoskeleton remodeling_Cytoskeleton remodeling	2.38 × 10^−10^
Glycolysis and gluconeogenesis (short map)	1.29 × 10^−9^
Cytoskeleton remodeling_Hyaluronic acid/ CD44 signaling pathways	4.43 × 10^−8^
Cytoskeleton remodeling_TGF, WNT and cytoskeletal remodeling	6.59 × 10^−8^
**Calu-1**	***p* Value**
Glycolysis and gluconeogenesis (short map)	7.76 × 10^−7^
Regulation of degradation of deltaF508-CFTR in CF	2.04 × 10^−6^
Transcription_Role of Akt in hypoxia induced HIF1 activation	3.03 × 10^−6^
LRRK2 and immune function in Parkinson's disease	1.88 × 10^−5^
Glycolysis and gluconeogenesis p.3	2.96 × 10^−5^
**Calu-6**	***p* Value**
Cytoskeleton remodeling_Cytoskeleton remodeling	4.86 × 10^−8^
CFTR folding and maturation (normal and CF)	5.09 × 10^−8^
Cell adhesion_PLAU signaling	1.11 × 10^−7^
Development_VEGF signaling via VEGFR2—generic cascades	5.96 × 10^−7^
Development_EGFR signaling pathway	1.3 × 10^−6^
**H157**	***p* Value**
LRRK2 in neurons in Parkinson‘s disease	2.21 × 10^−13^
Neurophysiological process_Receptor-mediated axon growth repulsion	6.45 × 10^−13^
Cytoskeleton remodeling_Cytoskeleton remodeling	3.35 × 10^−11^
Development_Slit-Robo signaling	6.54 × 10^−11^
Cytoskeleton remodeling_Regulation of actin cytoskeleton by Rho GTPases	1.14 × 10^−10^

**Table 2:** Altered kinase peptides by both MEK inhibitors in more than two cell lines.

Gene Symbol	Position	Cell Line	Direction of Change
ABL2	446	A427, Calu-1	Increase
AURKA	258	Calu-6, Calu-1, H157	Decrease
CHUK	146	Calu-6, H157	Decrease
CMPK1	16	Calu-6, H157	Decrease
CSNK2A2	159	A427, H157	Increase
JAK1	718	A549, Calu-1	Increase
LATS2	793	A549, A427, Calu-1	Increase
LATS2	697	A549, A427	Increase
MAP2K2	108	A549, Calu-6	Increase
MAP2K6	181	Calu-6, A427	Increase
MAST3	492	A427, H157	Increase
NADK2	76	Calu-6, A427, Calu-1	Increase
NEK2	143	Calu-6, H157	Decrease
NEK3	131	Calu-6, H157	Decrease
PGK1	184	A549, A427	Increase
PGK1	91	A427, Calu-1	Increase
PKM	322	A427, H157	Increase
PKM	66	A549, A427	Increase
PLK1	178	A549, Calu-6, Calu-1	Decrease
PLK1	82	A549, H157	Decrease
PRKAA1	40	A427, Calu-1	Increase
PRPF4B	727	A549, Calu-1	Increase
ROCK2	1065	A427, Calu-1	Increase
STK38	118	A427, Calu-1, H157	Increase
STK38L	119	A427, Calu-1	Increase
TK1	32	Calu-6, Calu-1	Decrease
TLK1	485	Calu-6, Calu-1	Increase
ULK1	140	A549, Calu-1	Increase

## Supplementary Materials

The following are available online at www.mdpi.com/2227-7382/4/2/16/s1: Table S1. Full list of ATP-binding proteins identified and quantitated on individual cell lines. Table S2. Full list of altered ATP-binding proteins on individual cell lines. Table S3. Full list of enriched pathways from altered ATP-binding proteins on individual cell lines. Table S4. Full list of kinases identified and quantitated on individual cell lines. Table S5. Full list of altered kinases on individual cell lines.

## Abbreviations

The following abbreviations are used in this manuscript:

IRON	Iterative rank-order normalization
LC-MS/MS	Liquid chromatography/tandem mass spectrometry
NSCLC	Non-small cell lung cancer
PCA	Principal component analysis
SAPK	Stress-activated protein kinase
